# Leveraging big data for causal understanding in mental health: a research framework

**DOI:** 10.3389/fpsyt.2024.1337740

**Published:** 2024-02-19

**Authors:** Jennifer J. Newson, Jerzy Bala, Jay N. Giedd, Benjamin Maxwell, Tara C. Thiagarajan

**Affiliations:** ^1^ Sapien Labs, Arlington, VA, United States; ^2^ Department of Psychiatry, University of California, San Diego, La Jolla, CA, United States; ^3^ Rady Children’s Hospital – San Diego, San Diego, CA, United States

**Keywords:** big data, mental health, MHQ, ABCD, Global Mind Project, machine learning, AI, causal factors

## Abstract

Over the past 30 years there have been numerous large-scale and longitudinal psychiatric research efforts to improve our understanding and treatment of mental health conditions. However, despite the huge effort by the research community and considerable funding, we still lack a causal understanding of most mental health disorders. Consequently, the majority of psychiatric diagnosis and treatment still operates at the level of symptomatic experience, rather than measuring or addressing root causes. This results in a trial-and-error approach that is a poor fit to underlying causality with poor clinical outcomes. Here we discuss how a research framework that originates from exploration of causal factors, rather than symptom groupings, applied to large scale multi-dimensional data can help address some of the current challenges facing mental health research and, in turn, clinical outcomes. Firstly, we describe some of the challenges and complexities underpinning the search for causal drivers of mental health conditions, focusing on current approaches to the assessment and diagnosis of psychiatric disorders, the many-to-many mappings between symptoms and causes, the search for biomarkers of heterogeneous symptom groups, and the multiple, dynamically interacting variables that influence our psychology. Secondly, we put forward a causal-orientated framework in the context of two large-scale datasets arising from the Adolescent Brain Cognitive Development (ABCD) study, the largest long-term study of brain development and child health in the United States, and the Global Mind Project which is the largest database in the world of mental health profiles along with life context information from 1.4 million people across the globe. Finally, we describe how analytical and machine learning approaches such as clustering and causal inference can be used on datasets such as these to help elucidate a more causal understanding of mental health conditions to enable diagnostic approaches and preventative solutions that tackle mental health challenges at their root cause.

## 1 Introduction

In the last three decades there have been many large-scale and longitudinal research initiatives aimed at enhancing our knowledge of mental health disorders and refining their treatment. Collectively, endeavors such as the Global Burden of Disease study (1), World Mental Health Surveys (2) and Psychiatric Genomics Consortium (3) have documented the prevalence of different mental health symptoms and their associated disorders; expanded our understanding of potential risk factors; and given us a better understanding of the complex genomic underpinnings that may result in symptoms associated with disorders such as bipolar disorder, depression, and schizophrenia.

However, despite intensive effort by the research community and considerable funding, a causal understanding of most mental health disorders remains elusive (4) and the majority of psychiatric diagnosis and treatment still operates at the level of symptomatic experience, rather than addressing root causes. This is analogous, within the domain of physical health, to physicians selecting treatments for conditions such as pneumonia, Covid-19, cancer, heart disease or diabetes based solely on a patient’s symptoms and sensations such as fever, pain, or fatigue, without having the necessary diagnostic or screening tests to know what’s caused them or what’s going on at a biological level. Furthermore, we know from physical conditions that the mapping between cause and symptom is typically a many-to-many mapping whereby the same constellation of symptoms can arise from multiple different causes, and the same set of causes can result in different constellations of symptoms across individuals. Having to diagnose and treat disorders based on symptoms alone therefore risks a trial-and-error approach that is a poor fit to underlying causality and results in poor clinical outcomes (5). Furthermore, within the field of psychiatry, diagnostic criteria for different disorders are theoretical constructs that are neither validated against underlying biology or cause, nor empirically demonstrated as separable symptom clusters (6, 7). Thus, the way symptoms are grouped within this system of disorder labeling may be grossly mismatched with underlying causality (7–9). Such mismatch creates substantial confusion in upstream efforts to identify treatments and biomarkers.

As a result of these challenges, progress in psychiatry lags behind many other medical specialties (10–14) and clinical outcomes for many patients remain poor (15–17). For example, an analysis of 102 meta-analyses covering 3,782 randomized clinical trials (RCTs) from over 650,000 participants, spanning most major mental disorders concluded “After more than half a century of research, thousands of RCTs and millions of invested funds, the effect sizes of psychotherapies and pharmacotherapies for mental disorders are limited” (18). In addition, the prevalence of suicide and mental health symptoms is high, and on the rise (19, 20) with suicide remaining the fourth leading cause of death among 15-29 year-olds globally (20) and the 2^nd^ leading cause of death for people aged 10-14 and 20-34 in the United States (US) (21). In addition, the prevalence of depression and anxiety in young people has steadily increased, exacerbated by the Covid-19 pandemic (22–24). This latter finding is visible as a striking shift in mental wellbeing trends across age groups, where, in the early 2000s, studies showed that young adults (ages 18-21) had the highest psychological wellbeing dipping in middle age and rising again in older age groups, a phenomenon that came to be known as the U-shaped curve of happiness (25). However, since 2011, the Centre for Disease Prevention (CDC) has shown that, in the US, younger age groups increasingly express feelings of sadness (19), while a trend of diminishing mental wellbeing in young people is observed on virtually every continent (26). It is also significant that the overall burden of psychiatric disease is greater in western English-speaking countries despite greater per capita income, a larger number of psychiatrists per population and mental health spending that is 5-7 times higher than other countries with lower incidence levels (1, 27, 28).

However, recent advances in large-scale data acquisition, open datasets and analytical/machine learning approaches present a new era of opportunity within mental health research (4, 29–32) to deal with the multitude of biological, social and environmental factors which can influence the brain and mental health and unpack their complex relationships. This allows a refocus of the mental health research paradigm to deliver a more coherent understanding of both the causal factors and physiological underpinnings of psychiatric conditions to enable better prevention, diagnosis and treatment.

In this paper, we propose a shift in the existing paradigm of mental health research from one that starts with theoretically defined categorical symptom groupings to one that embraces a multidimensional approach using large datasets to develop testable causal hypotheses. To that end, we discuss the challenges and complexities associated with uncovering underlying causes of mental illness, focusing on current diagnostic frameworks, the many-to-many mapping between cause and symptoms, and the interplay between root causes, physiological markers and symptomatic experience. We then describe two large-scale multi-dimensional data projects, the Adolescent Brain Cognitive Development (ABCD) Study and Global Mind Project and discuss how machine learning approaches applied to datasets such as these can aid in identifying causal factors and help psychiatry take a much-needed leap forward.

## 2 Challenges and complexities of identifying causal drivers of mental illness

### 2.1 Current diagnostic frameworks preclude a causal understanding

Historically, the classification of psychiatric disorders has been driven by clinical observations combined with a theoretical framework that groups symptoms into diagnostic criteria. These criteria are laid out in manuals such as the Diagnostic and Statistical Manual of Mental Disorders (DSM-5) (33) and the International Classification of Diseases (ICD-11) (34) and are used by clinicians to assign patients to particular diagnostic labels based on the alignment of their particular symptom profile to diagnostic criteria (e.g. Major depression: 5 or more depressive symptoms for ≥ 2 weeks; must have either depressed mood or loss of interest/pleasure). In turn, this guides their treatment and care management pathway, helps with ease of communication and documentation, and, in countries such as the US, determines the amount of health insurance support that patients will receive. In the context of research, these diagnostic categories are also used to determine how patients are selected for, and allocated to, different experimental groups, particularly in clinical trials.

In an ideal world, a robust diagnostic system should have high sensitivity to correctly identify patients who have a particular condition, and high specificity to correctly exclude individuals who do not have the condition. In addition, the definition of this condition should have biological validity, correlating with neuroimaging, genetic, or other biomarkers. However, despite multiple iterations of these classification manuals over the past few decades, the current system of classifying individuals based on symptom criteria does not meet this ideal (6, 35, 36) and encounters several challenges that hinder the establishment of a causal understanding.

First, these symptom-based disorder classifications often share similar symptoms within their criteria, making it difficult to distinguish between them. For example, impaired sleep is common to several disorders including attention-deficit/hyperactivity disorder (ADHD), anxiety disorder, autism spectrum disorder (ASD), mood disorders, substance use disorder, and post-traumatic stress disorder (PTSD) (37–39). Consequently, it is common for patients to be comorbid across multiple disorders, rather than having symptoms that only align with one disorder (40, 41). Second, there is substantial heterogeneity within diagnostic categories, where individuals with the same diagnostic label can have diverse symptom profiles and treatment needs. For example, there are over a hundred different symptom combinations that can lead to a diagnosis of depression, ADHD or PTSD (42–44). Third, it is common for a patient’s symptom profile to evolve and shift over time, crisscrossing different disorder categories, especially within child and adolescent psychiatry where developmental factors create a moving target of symptoms (45–47).

This mismatch between symptomatic experience and disorder classifications was evidenced in a recent study of over 100,000 individuals that showed that the heterogeneity of their symptom profiles was almost as high within a psychiatric disorder category as between any two disorder categories (6). Furthermore, no individual disorder category was separable from randomly selected groups of individuals with at least one disorder, indicating that DSM-5 disorder criteria failed to separate individuals by symptom profiles any better than random assignment.

Altogether, the dominance of theoretical classification frameworks based on symptom groupings that are difficult to distinguish from one another and not tied to underlying causes, disrupts the search for linkages between symptoms and their root causes. New transdiagnostic frameworks have emerged such as Research Domain Criteria (RDoC) (7, 48, 49) put forward by the National Institute of Mental Health (NIMH) that considers mental health and psychopathology in the context of major functional neuroscientific domains (e.g. cognition, social processes, sensorimotor, positive/negative arousal, regulation). However, while this framework focuses on functional classifications and physiological criteria, it also does not provide a method of linking causes to symptoms presented in clinical practice.

### 2.2 Causes to symptoms have a many-to-many mapping

Another challenge that hinders the identification of causal drivers of mental illness is the complex interplay between cause and symptoms. To illustrate this complexity, we provide here an analogy to the physical illness of Covid-19. Covid-19 is a viral infection caused by SARS-CoV-2 with the likelihood of infection dependent on a whole host of secondary biological and social contributing factors. The constellation of symptoms it evokes in people is highly heterogeneous and can include anything from cough, cold, fever and breathing difficulty to fatigue, chills, headaches and brain fog, while some people can be asymptomatic. Conversely, the same constellation of symptoms can be evoked by other causal agents such as bacterial infections, fungal infections, poisons and toxins, poor diet or smoking. Therefore, there is not a 1-to-1 mapping between cause and symptoms, but instead a many-to-many mapping (**Table 1**).

**Table 1:** Example of many-to-many mapping between cause and symptoms.

Causes	Symptoms
Viral Infections (Flu, COVID-19, other) Bacterial Infections Fungal infections Amoeba infections Some worm infections Certain Toxins Smoking Poor diet Physical trauma	Fever Body Ache Fatigue Chills Headache Cough Congestion/Runny nose Difficulty breathing Diarrhea Brain fog

If the symptom-based approach of psychiatry was applied in this context, then the physical symptoms that often tend to present together (cough, cold, fever, sore throat, and breathing difficulty to fatigue, muscle weakness, chills and headaches etc.) might be, in aggregate, labeled “Body Depression Disorder” where having at least 3 or 4 of these symptoms may qualify you for the diagnosis. However, it would be impossible to identify the specific cause (e.g. Covid-19 vs worms vs poor diet) and individuals who were not responsive to a commonly prescribed medication for “Body Depression Disorder” such as an antibiotic, may simply be considered ‘treatment resistant’.

Within the domain of mental illness, this same many-to-many mapping applies between symptoms and root causes. For example, pathogens such as syphilis and streptococcus (if they cross the blood-brain barrier) have been shown to evoke a set of symptoms that align with the diagnostic criteria associated with the disorder labels of schizophrenia and obsessive compulsive disorder (OCD), respectively (50, 51). In turn, these disorder labels, have also been associated with multiple other causal factors and heterogeneous symptom and physiological profiles (52–54). Similarly, the symptom-based diagnosis of depression has been associated with a host of environmental factors including ultra-processed food consumption, traumatic experiences and brain injury (55–57), while individuals given a diagnosis of depression can exhibit highly heterogeneous symptom profiles beyond the diagnostic criteria outlined in DSM-5 (6, 58). Altogether, mental illness has a greater range of potential causes extending beyond pathogens, toxins and injury to include social experience and sensory stimulus.

**Figure 1:**
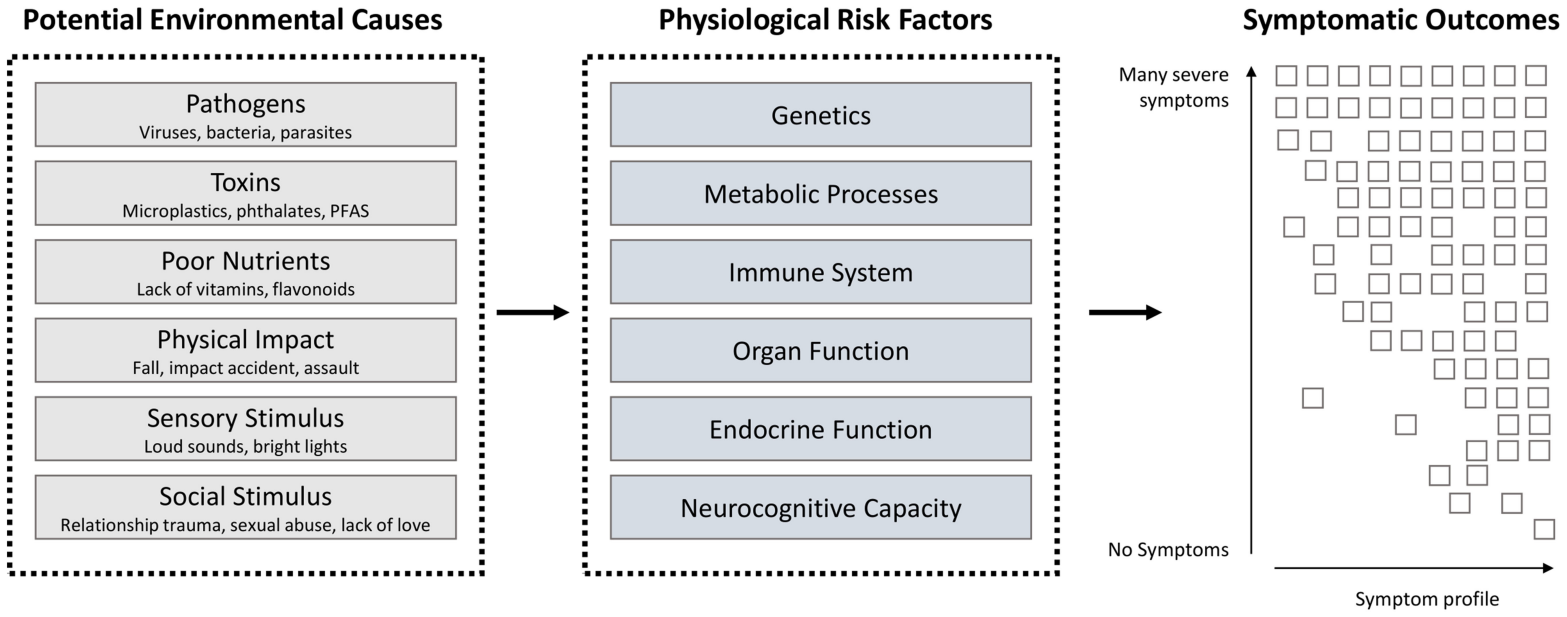
Various environmental assaults interact with the physiology of the individual to produce diverse symptomatic outcomes (individual symptoms represented by boxes). For example, a pathogen such as SARS-CoV-2 interacts with the immune system and various organs to produce anything from no symptoms to very severe symptoms of different types such as breathing difficulty or extreme fatigue and high fever. A broader range of environmental exposures as shown above can interact with the body to deliver a wide range of mental symptoms.

Compounding this challenge is that the emergence of symptoms from these causal assaults depends critically on the physiology and genetics of the individual (**Figure 1**). In the case of COVID-19, those with immune compromise or obesity are more likely to experience a broader array of severe symptoms while those who are young and healthy may go entirely asymptomatic (59). So also, a traumatic experience or diet profile could result in very different mental health outcomes depending on individual physiology or genetics (60, 61). Thus, no perfect relationship exists for any cause and symptom combination, and even less so for any theoretically defined symptom grouping. This illustrates why traditional experimental approaches that are set up to evaluate differences between a symptom-based diagnostic group and healthy controls have not been very successful, resulting in considerable debate around treatment efficacy (e.g. for antidepressants (62–64)). It also highlights the need for a multi-dimensional approach which considers a range of symptoms, physiological underpinnings and causal factors from the outset.

### 2.3 Biomarkers of symptoms can be misleading

The search for biomarkers of mental health disorders has been an active area of investigation (65). However, despite decades of research there are still no biomarkers that form a crucial part of accepted diagnosis (8, 66, 67). Why is this so? One reason is that mental disorders are highly heterogeneous groupings of symptoms with multiple potential causes so no single physiological marker can be definitive, as it will likely apply only to a subset of those with the symptom-defined diagnosis. Consequently, none have passed a threshold of accepted statistical significance. A second aspect, however, is that biomarkers of symptoms can be misleading.

To illustrate this, we return again to the example of “Body Depression Disorder” which we laid out above. One may find that elevated white blood cell (WBC) counts are fairly reliably associated with a diagnosis of “Body Depression Disorder”. However, the subset of those with injury or physical trauma which still aligns with the criteria for “Body Depression Disorder” would not be associated with this WBC biomarker. Similarly, one might find a reduced electromyography (EMG) signal (a measure of the strength of muscle contraction) is commonly associated with “Body Depression Disorder” although it may be more reliably associated with a particular constellation of sub-symptoms, (e.g., muscle weakness, fatigue, and fever) rather than the diagnosis in general. While these markers may indicate a physiological challenge, they do not necessarily inform treatment. If the function of WBCs was not well understood, it would be tempting to declare WBCs as the cause of “Body Depression Disorder” and thereby seek treatments that eliminate or manipulate levels of WBCs. Alternatively, if one considered that a weak EMG was the cause, then one might electrically stimulate muscles in the hope that it will spur them into action. Thus, while these “biomarkers” act as reasonably good predictors of the symptoms, targeting these factors as a treatment pathway would be a grave mistake.

When considered in the context of mental illness, with the absence of causal understanding, we may find that specific metabolites in blood or cerebrospinal fluid (CSF), or particular physiological characteristics within a single-photon emission computed tomography (SPECT) scan or electroencephalography (EEG) readout are predictive of symptom subsets. However, they may be an indirect biomarker of those symptoms (much like WBCs) rather than the direct cause of them, and therefore targeting them for treatment would not be appropriate. Instead, a more useful biomarker would be an assay for the causal factor itself (analogous to an antigen test for Covid-19) which can in turn inform both prevention and treatment at the causal level.

**Figure 2:**
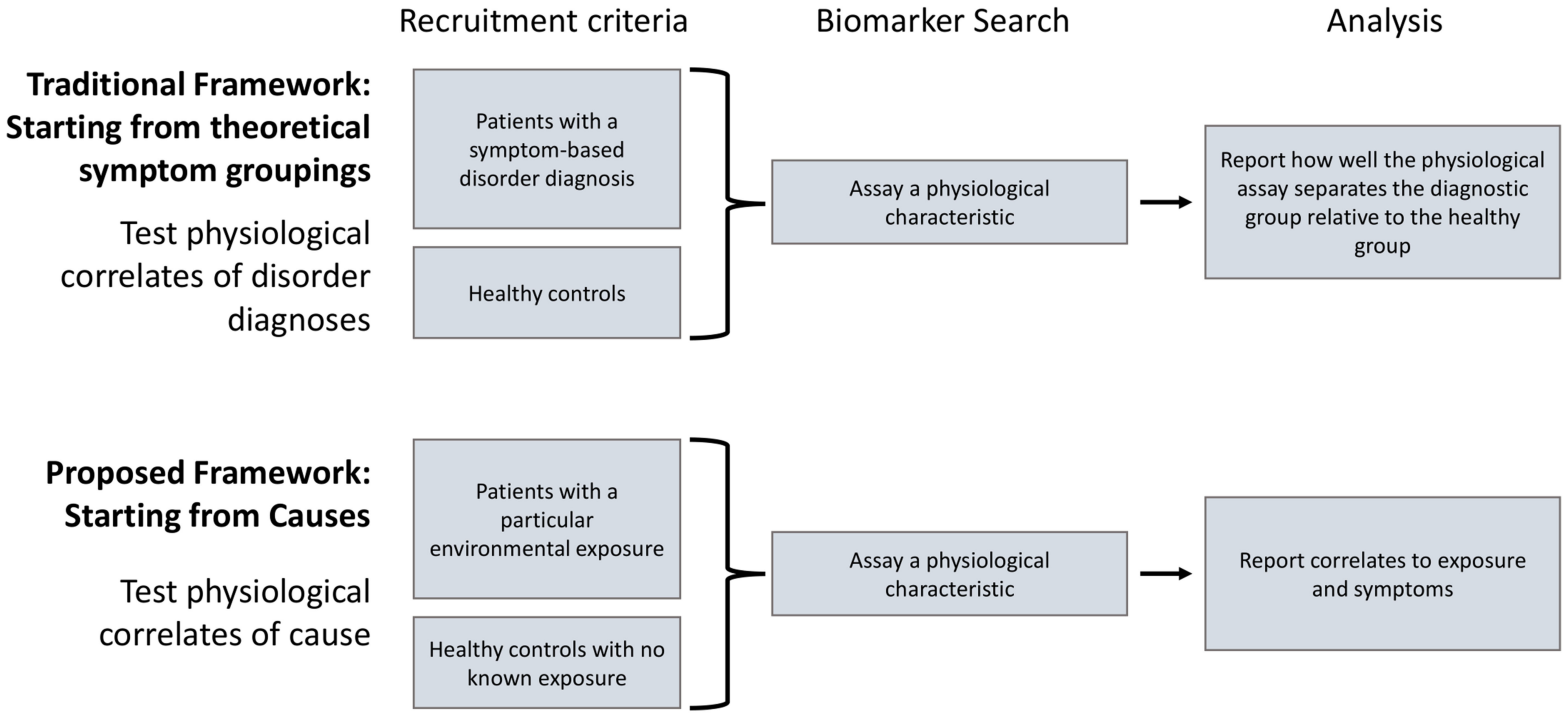
The current paradigm involves searching for biomarkers as physiological correlates of symptom-based disorder definitions (top). As these disorder definitions are likely to have multiple potential causes, and encompass highly heterogeneous symptom profiles, we propose a shift toward identifying biomarkers that are physiological correlates of both causal factors and specific symptoms (bottom).

We thus propose a shift in the framework from searching for biomarkers of symptoms to searching for biomarkers of causes that can serve as potential diagnostic criteria, and potential targets for treatments (**Figure 2**).

## 3 Strategies for a causal-oriented research framework

### 3.1 Developing a multidimensional approach to generate testable hypotheses

**Figure 3:**
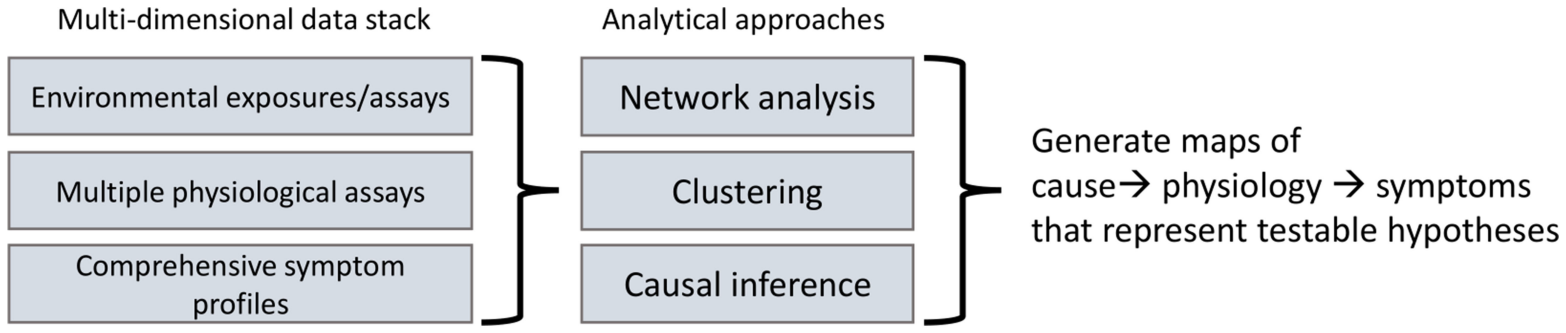
Generating causal hypotheses in observational data: Moving toward a causal diagnostic framework requires generating testable causal hypotheses from large-scale multidimensional observational data consisting of environmental exposures, physiological assays (e.g., from blood, urine, saliva) along with comprehensive symptom profiles and analyzing these using various multivariate techniques such as causal inference to generate maps of cause to physiology to symptoms.

Considering the broad range of biological, social, and environmental factors that may be at play, moving from a categorical symptom framework of diagnosis to a causal one requires researchers to initially cast a wide net to explore multiple possibilities of cause in order to generate testable hypotheses and identify candidate biomarkers (**Figure 3**). From the perspective of mental illness this includes not only pathogens and a host of chemical toxins but also aspects of the stimulus environment such as social and technological experiences and lifestyle.

Secondly, these causal possibilities must be considered in relation to comprehensive individual symptom profiles rather than being limited to theoretical groupings of symptoms categorized as pre-defined disorders. This approach can then identify well-substantiated hypotheses that can be tested through various methods.

Finally, the inclusion of measures of physiology and neurophysiology can aid in the refinement of such hypotheses and the discovery of diagnostic biomarkers. For example, blood markers could help distinguish between the same symptoms caused by pathogens and toxins versus by injury or traumatic experience.

Achieving this requires a shift away from the common approach of comparing a single causal possibility for pre-determined symptom-based diagnostic groups, to a multidimensional approach using large datasets. Such datasets should include comprehensive symptom profiles, assessment of a wide range of potential causal factors and physiological readouts, coupled with analytical approaches amenable to untangling the complex relationships of variables. With new tools for both data acquisition and analysis we are now entering an era that makes this possible.

### 3.2 The big data opportunity

Over the years, psychiatry research has faced limitations in both acquiring and handling vast amounts of data to explore a wide breadth of variables. Fortunately, recent advancements in data science have opened new doors for transformative progress in the field. Here we talk about two substantially different ongoing large-scale data acquisition efforts, and the potential they have in moving the field toward a causal diagnostic framework: the Adolescent Brain Cognitive Development (ABCD) Study and the Global Mind Project. Briefly, the ABCD study follows 11,864 young people in the US recruited at ages 9 or 10 into adulthood, annually characterizing various potentially causal aspects of their environment and their mental health status along with neuroimaging and genetic studies (**Table 2**). The Global Mind Project dynamically tracks detailed mental health symptom profiles of individuals around the globe along with demographic information and various social, technological, and environmental factors that are potential causes of mental health challenges. Since 2020 the project has collected responses from over 1.4 million people 18 years and older across 70+ countries in 12+ languages.

**Table 2:** A summary of the ABCD Study and Global Mind Project.

Project	Size	Type	Population	Data Types
ABCD Study	11,000 +	Longitudinal	US only Ages 9-20	Psychiatric and Neurocognitive assessments, Physiological assays Environmental surveys
Global Mind Project	1.4 Million +	Realtime snapshot	70+ countries Ages 18+	Comprehensive symptom profiles, Extensive environmental survey

### 3.3 ABCD Study^®^

The ABCD Study^®^ is a 21-site US-based project integrating longitudinal neuroimaging, genetics and behavioral assessments of 11,864 youth and has been described in detail in multiple publications (68–73). Youth were recruited into the study beginning at age 9 or 10 in 2015 and are tracked along various dimensions through their childhood with the goal of understanding how social, behavioral, physical, and environmental factors affect brain development and other health outcomes through the second decade of life. The earliest cohort are now in their 9^th^ year of assessment. Study assessments are conducted annually or biannually and include an extensive neurocognitive battery and psychological/behavioral assessments covering various disorders as well as questionnaires on family history and structure, substance use history and screen time (**Table 3**) (74, 78). In addition, the study includes Magnetic Resonance Imaging (MRI) at two-year intervals (structural imaging, diffusion tensor imaging (DTI), and task-based and resting-state functional imaging) (71). Biospecimens are collected annually and include hair samples, deciduous baby teeth, and body fluids (blood, saliva and urine) to assess exposure to illicit and recreational drugs, pubertal hormones, genomics and epigenomics, pre- and post-natal exposure to environmental neurotoxicants and drugs of abuse (77). The total number of survey questions is approximately 1200 (depending on the number of answers that trigger additional questions) although many of the questions in the psychological surveys overlap due to the large overlap of symptoms across disorder specific tools. Importantly, the ABCD Study is a longitudinal study which allows not just snapshot views but the ability to look temporally at the trajectory of symptoms.

**Table 3:** ABCD Study assessments.

	Assessment type	List of Surveys/Tasks
Mental health and cognitive outcomes	Psychological surveys (74)	Kiddie Schedule for Affective Disorders and Schizophrenia (KSADS-5) [Youth and Parent for Background items and DSM-5 Diagnostic Interview]; UPPS-P for Children - Short Form (ABCD version) [impulsivity]; Behavioral Inhibition/Behavioral Approach System (BIS/BAS) Scales [Inhibition and reward seeking]; Prodromal Psychosis Scale [Prodromal psychosis level]; Youth Resilience Scale Resilience [friends]; Child Behavior Checklist [Dimensional psychopathology, adaptive functioning]; Parent General Behavior Inventory - Mania Subsyndromal [mania]; Adult Self Report Parent [Dimensional psychopathology]; Family History Assessment [for biological or adoptive parent]; and 5 Substance Abuse Questionnaires [Participant Last Use Survey (PLUS) for substance use within the last 24 hours; PhenX* Peer Group Deviance Survey; PATH Intention to Use Tobacco Survey; Timeline Follow-Back Survey; and Caffeine Intake Survey].
	Neurocognitive tasks (75)	Rey Auditory Verbal Learning Task; Cash Choice Task; Little Man Task; Matrix Reasoning Task; and RAVLT Delayed Recall.
	NIH Toolbox tasks^&^ (75)	Picture Vocabulary; Flanker Inhibitory Control & Attention; List Sorting Working Memory; Dimensional Change Card Sort; Pattern Comparison Processing Speed; Picture Sequence Memory; Oral Reading Recognition.
Potential causal and risk factors	Cultural and environmental surveys (74)	Prosocial Behavior Survey; PhenX* Acculturation Survey; Parental Monitoring Survey; Acceptance Subscale from Children’s Report of Parental Behavior Inventory – Short; PhenX* Family Environment Scale - Family Conflict; PhenX* Neighborhood Safety/Crime Survey; PhenX* School Risk & Protective Factors Survey; and Screen Time Survey.
	Linked External Data (LED) (76)	EPA smart location database, FBI Uniform Crime Report, American Community Survey Area Deprivation Index, Elevation from Google Maps, and NASA Socioeconomic Data and Applications
Physiological readouts of outcomes	Functional MRI (71)	Monetary Incentive Delay Task; Stop Signal Task; Emotional N-Back Task. Resting state.
	Structural MRI (71)	High resolution 3D T1 – and T2 – weighted, Diffusion Tensor Imaging
	Biospecimens (77) Hair, baby teeth, blood, saliva, urine, hair	Assays for exposure to illicit and recreational drugs, pubertal hormones, genomics and epigenomics, pre- and post-natal exposure to environmental neurotoxicants and drugs of abuse

* https://www.phenxtoolkit.org/.

^&^ https://www.nihtoolbox.org/.

The ABCD data is curated and accessible via the NIMH Data Archive and released annually. The ABCD Study’s Data Release 5.0 is now available (https://dx.doi.org/10.15154/8873-zj65), https://abcdstudy.org/. Only researchers with an approved NDA Data Use Certification (DUC) may obtain ABCD Study data. It requires verification through one of three NIH Auth Service (RAS) identities.

While the data includes recruitment across 21 sites with wide socio-economic and ethnic representation, it has a unique set of challenges and potential biases given its longitudinal nature. These include attrition over time and data gaps due to suspension of certain aspects of data collection during the Covid-19 pandemic. More specifics on the ABCD data and these potential biases are described in Saragosa-Harris et al. (73).

#### 3.3.1 Key findings of interest

Thus far, major findings reported from the ABCD Study have regarded the potentially causal impact of genetics, sleep, exercise, music, nutrition, trauma, and social media use on brain structure and function, particularly regarding executive functions such as planning, decision making, and impulse control (79, 80). These functions are generally subserved by neural circuitry involving the prefrontal cortex which is known to be dynamically developing well into the third decade of life (81). Some specific findings of public health interest have included:

*The negative impact of recreational screen use in adolescents*. Data from the ABCD Study have added to a growing body of literature highlighting the negative association between screen use and cognitive and mental health outcomes in youth (80, 82). Research has also revealed the impacts of screen use on sleep, showing that screen use (television or interconnected devices) at bedtime was significantly associated with sleep disturbances in children aged 11-12 (83).

*The relationship between sleep quality, neurocognitive development, and mental health symptoms.* To date, findings have shown how sleep quality and duration are robustly associated with neurocognitive development, mental health symptoms, and brain anatomy/physiology in children and adolescents (84, 85). In particular, children with shorter sleep duration have smaller brain volumes in areas related to cognition and higher psychiatric problems scores (as do their parents) (84). In addition, insufficient sleep (defined as < 9 hours) has been shown to have widespread effects on baseline behavioral and functional connectivity measures (85).

However, thus far, most studies have focused on the relationships between specific environmental factors and outcomes. This leaves open a vast opportunity for multidimensional analysis that identifies the relative contributions of different environmental factors to both physiological and mental health outcomes and the consequences of their interactions. We outline some possible approaches in Section 4.

### 3.4 Global Mind Project

The Global Mind Project, launched in 2020, dynamically tracks detailed mental wellbeing profiles of individuals around the globe along with demographic information and various social, technological, and environmental factors that are potential causes of mental health challenges. Since inception, the project has collected responses from over 1.4 million people 18 years and older across 70+ countries in 12+ languages (**Table 2**). Approximately 1000-2000 new responses are added per day across diverse demographics. More recently data from ages 13 to 17 have also been included.

The study uses a transdiagnostic assessment of mental wellbeing, called the Mental Health Quotient (MHQ) that is completed online and collects life impact ratings across 47 different elements of mental feeling and function spanning all possible symptoms of 10 major mental health disorders, as well as positive aspects of functioning. In addition, the assessment captures detailed demographics, information on various aspects of the social and technology environment as well as lifestyle factors. Data is collected through inviting participation through online advertising that targets a broad range of demographics in each country. The sample is thus specific to the internet-enabled populations of each country. The US sample, where internet penetration is over 90%, has been shown to be broadly representative of the national population, closely matching various demographic and mental health patterns in the American Community and Household Pulse Surveys conducted by the US Census Bureau (86).

Additionally, the dynamic nature of the Global Mind Project offers a view of the ongoing evolution of mental wellbeing and the agility to quickly probe new potential causal factors at scale to understand the impact of emerging social and environmental factors. A summary of the demographic and potential causal factors considered thus far are shown in **Table 4** below.

**Table 4:** Global Mind Project data elements.

MHQ Stats	Number	Type
Mental health outcomes	47	Spans symptoms associated with 10 disorders as defined by DSM-5: depression, anxiety, bipolar disorder, PTSD, ASD, ADHD, psychosis, addiction, OCD, and eating disorder. Also includes elements from the RDoC framework and dementia diagnosis (27 mental functions with positive and negative dimensions, 20 problems).
Traumas/adversities	22	Life threatening or debilitating injury or illness; Sudden or premature death of a parent or sibling/Sudden or premature death of a loved one; Parental divorce or family breakup/Divorce/separation or family breakup; Prolonged physical abuse, or severe physical assault; Prolonged sexual abuse, or severe sexual assault; Physical violence in the home between family members (e.g. between parents); Cyberbullying or online abuse; Prolonged or sustained bullying in person from peers; Prolonged emotional or psychological abuse or neglect from parent/caregiver; Lived with a parent/caregiver who was an alcoholic or who regularly used street drugs; Extreme poverty leading to homelessness and/or hunger; Involvement or close witness to a war; Displacement from your home due to political, environmental or economic reasons; Serious injury, harm, or death you caused to someone else; Suffered a loss in a major fire, flood, earthquake, or natural disaster; Threatening, coercive or controlling behavior by another person; Forced family control over major life decisions (e.g. marriage); Caring for a parent or sibling with a major chronic disability or illness/Caring for a child or partner with a major chronic disability or illness; Parent/Caregiver/Sibling with mental illness or who committed suicide; Parent/Caregiver/Sibling went to prison; Loss of your job or livelihood leading to an inability to make ends meet.
Demographic factors	11	Age; Biological sex; Gender; Country; State; Rural/Urban; City; Race/Ethnicity; Education/Employment; Profession; Household income
Lifestyle factors	5	Sleep; In person socializing; Physical exercise; Processed food consumption; Substance use
Social factors	18	Number of people share house with; Family situation; Number of children; Number of siblings growing up; Number of close friends; Number of childhood friends; Proximity of close friends; Friends to help out; Friends to confide in; Nature of household growing up; Parental/caregiver support; Relationship with adult family; Proximity to adult family; Spiritual connection; Feeling of love toward others; Religious identity; Religious practice; Individualism/Collectivism
Technology Factors	11	Age of first smartphone ownership; Age of first tablet ownership; Friend/classmate smartphone ownership; Internet restriction during childhood; Age of first smartphone use in school; Smartphone usage during class; Smartphone usage during recess; Age of first personal laptop/tablet use at school; Personal laptop/tablet use using class; Age of first social media account; Social media posting frequency
Health Factors	10	Mental health treatment status; Reasons for not help seeking; Treatment type; Effectiveness of treatment; Mental health diagnosis; Physical health condition; Covid-19 health & social adversities; Covid-19 financial adversities; Pregnancy; Physical complaints

The data are openly available in real-time to not-for-profit researchers in structured format and the data can be searched and downloaded by time period, country, language, age and gender. Access to the dynamically updated data is available through Sapien Labs’ proprietary platform Brainbase for which access must be requested through the request form at this url: https://sapienlabs.org/global-mind-project/researcher-hub/.

The MHQ assessment is comprehensive in its coverage of mental health symptoms, yet compact, which allows for more streamlined analysis of symptom profiles without the need to stitch together various assessments which are characterized by significant overlap of symptoms and a lack of standardization (87). The overall aggregate metric of mental wellbeing, dimensional scores, as well as individual ratings (1-9 life impact Likert scale) for 47 individual elements of feeling and functioning, also allows for outcome analysis at different levels of granularity.

#### 3.4.1 Key findings of interest

Thus far, the Global Mind Project has identified relationships between key potentially causal environmental factors and specific symptoms that are of public health interest:

*Age of first smartphone and adult mental health.* The data has shown that younger ages of first smartphone ownership in childhood are progressively associated with poorer mental wellbeing in adulthood and in particular a greater incidence of “Suicidal thoughts & intentions”, “Feelings of being detached from reality” and “Feelings of aggression toward others” in early adulthood, particularly for girls (88). This trend persists when controlling for childhood traumas and adversities.

*Ultra-processed food consumption and symptoms of depression and cognitive/emotional control.* Data from 300,000 people in 2023 showed that more frequent consumption of ultra-processed food is associated with significantly lower mental wellbeing, independent of differences in exercise frequency and household income (89). In particular, “Appetite regulation”, “Feelings of sadness, distress or hopelessness” as well as various challenges with emotional and cognitive control were most significantly increased with higher frequencies of ultra-processed food consumption.

The wide range of life context factors that are potentially causal also allows for a rich analysis of interactions. A recent multidimensional analysis that included multiple potential causal factors has used supervised learning to show that social behavior has a far greater impact on overall mental wellbeing outcomes in the population compared to exercise, traumas and adversities and substance use (90).

Altogether the Global Mind Project enables rapid generation of causal hypotheses as well as understanding of the hierarchy of impact of causal factors that can then be tested in follow-on studies.

## 4 Analytical approaches for causal understanding

The application of data science techniques to the large-scale datasets described above provides a powerful way to understand the many-to-many relationships between causes, symptoms and physiology. We present here examples of approaches that can be applied to the ABCD and Global Mind datasets.

### 4.1 Understanding groupings of symptoms and causes using clustering approaches

In contrast to the present symptom-based approach which groups symptoms theoretically, clustering or unsupervised learning approaches (91) can be used to determine if there are indeed empirically separable symptom groupings. Such empirically separable groups that map more strongly to specific physiological metrics and/or social or environmental factors could then suggest a specific underlying cause or disease grouping that can be more rigorously tested. Such symptom clustering can be easily achieved in the Global Mind dataset across a large and culturally diverse population where 47 symptoms are collected in a single assessment. While the ABCD Study queries symptoms across a number of different assessments which would have to be combined to construct a comprehensive symptom profile for each individual, it offers an opportunity to both determine how symptom clusters emerge during adolescence and how they might evolve over time.

**Figure 4:**
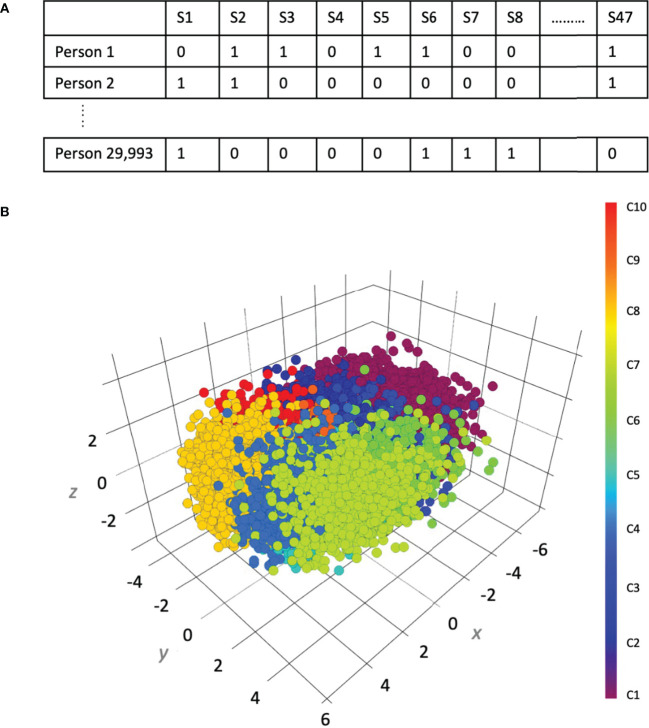
An example of clustering of symptom profiles using 29,993 records from the Global Mind data. **(A)** The construction of symptom profiles across 47 symptoms (columns) for 29,993 individuals (rows). **(B)** Uniform manifold approximation and projection of symptom clusters.

We show in **Figure 4** an example of clustering of symptom phenotypes using data from 29,993 people from the Global Mind data with five or more symptoms. Here, each individual either has or does not have each of 47 symptoms queried (**Figure 4A**) based on whether or not their rating crosses the threshold to be considered a symptom. A clustering algorithm then seeks to group individuals based on the similarity of their symptom profile. As one example, a 3-D projection of potential clusters using Principal Component Analysis (PCA) is shown in **Figure 4B** (92). Visually, there is poor separability of groupings overall which suggests that there are no clear symptom phenotypes. However, it is possible that on closer examination some clusters may separate better than others. Moreover, there are numerous approaches to clustering, as well as different levels at which the clustering can be performed, which may confer better separability. The first challenge is to determine the best way of computing similarity of symptom profiles. Drawing again the analogy to physical symptoms, fatigue may be a common symptom of almost all diseases whereas other symptoms such as a cough and cold can clearly restrict possible etiology to viral or bacterial pathogens. However, having many common symptoms like fatigue will reduce the separability of symptom profiles. Thus, understanding of the hierarchy of how symptoms behave can inform how one should approach clustering and which method(s) out of the many available should be implemented (see below for some examples). Conversely, the problem can be approached from the opposite direction where social and environmental factors can be clustered to identify life context phenotypes that map to particular symptoms and/or physiological phenotypes. The presence of empirically separable symptom clusters, especially if enriched for particular life context factors, could be substantially informative about the underlying cause.

Within the toolkit of machine learning there are many clustering approaches. For example, hierarchical clustering (93) organizes elements (such as symptoms) into a tree-like structure, which reveals both higher-level clusters and individual symptom relationships. On the other hand, K-Means clustering (94) creates a specified number of symptom groups by assigning each symptom to a cluster by minimizing the distance between symptoms within the same cluster while maximizing the distance between clusters. Other approaches include Density-Based Spatial Clustering of Applications with Noise (DBSCAN) (95), which dynamically determines the number of clusters based on the density of data points, as well as the Gaussian Mixture Model (GMM), which is a probabilistic model that accounts for overlapping symptom patterns (96).

Depending on the structure of the data, different clustering approaches can sometimes produce substantially different results (97, 98). Cluster validity and reliability can be affected by factors such as the distance metric used, the presence of outliers, and the distribution of the data. Additionally, dimensionality can pose a challenge, making techniques such as Principal Component Analysis (PCA) useful for preprocessing. Thus, multiple methods have to be explored and compared to identify robust and reproducible results.

### 4.2 Identifying the hierarchy of causal risk factors in symptom outcomes

**Figure 5:**
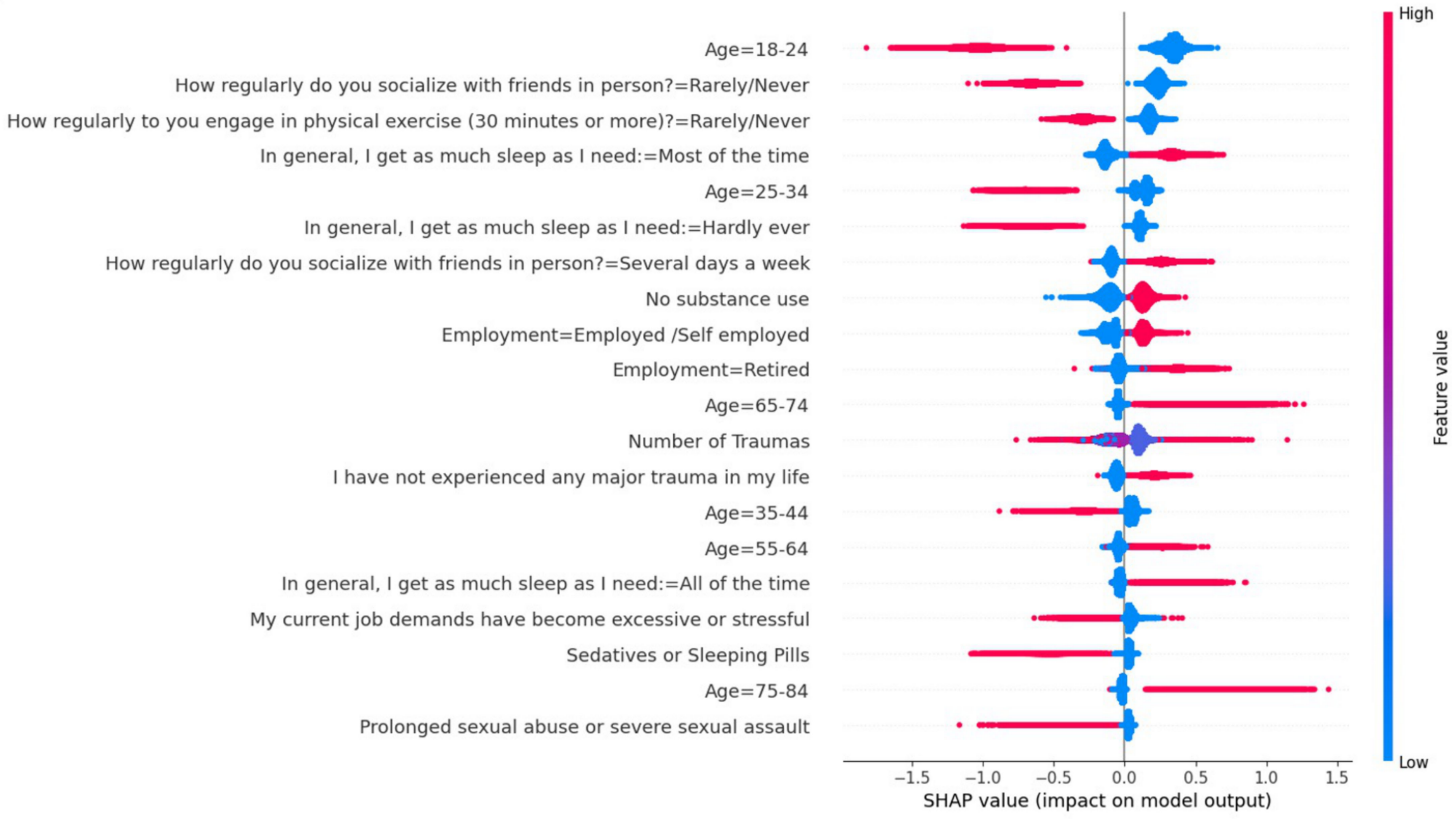
SHAP analysis of factors showing various demographic and lifestyle categories and their impact on the prediction of mental health status as determined by the MHQ score using a gradient boosting model. Red indicates a significant impact, while values to the left of 0 on the scale indicate that it contributes to a negative mental health status, and values to the right of 0 indicate a contribution to a positive mental health status.

One obvious application of large multidimensional data is the ability to identify the hierarchy of potential causal factors. While many factors may drive each symptom or grouping of symptoms, they may do so to different degrees. Various supervised learning approaches can be used to determine how combinations of potential causal factors, such as social determinants or physiological metrics, predict mental health outcomes. These include logistic regression (99, 100), gradient boosting (101, 102), random forest (103, 104) and naïve bayes (105), which are all described in detail in various machine learning textbooks and tutorials. As a corollary to such predictive models, different techniques can be used to determine how important each input is for the prediction. Such ranking can help determine which factors should be the focus of further research or intervention. In **Figure 5**, we show one such method called SHAP (106) applied to a gradient boosting prediction model (XGBoost), which uses a tree-based approach to prediction using 270,000 records from the Global Mind Project obtained in 2022. This example includes demographics as well as lifestyle factors demonstrating that certain factors contribute to negative mental wellbeing outcomes (negative MHQ outcomes) while others contribute to positive mental wellbeing outcomes. While this is for illustration purposes only, it is evident that various lifestyle and life context factors such as lack of social behavior and exercise as well as sleeping pills, job stress and sexual abuse contribute to negative mental health while good sleep, regular exercise and regular socializing contribute to positive mental health. In addition, age is a significant factor with younger age contributing to poorer mental health and posing the question of what type of causal factors associated with young age may be missing (for example, early age of smartphone ownership or social media use).

**Figure 6:**
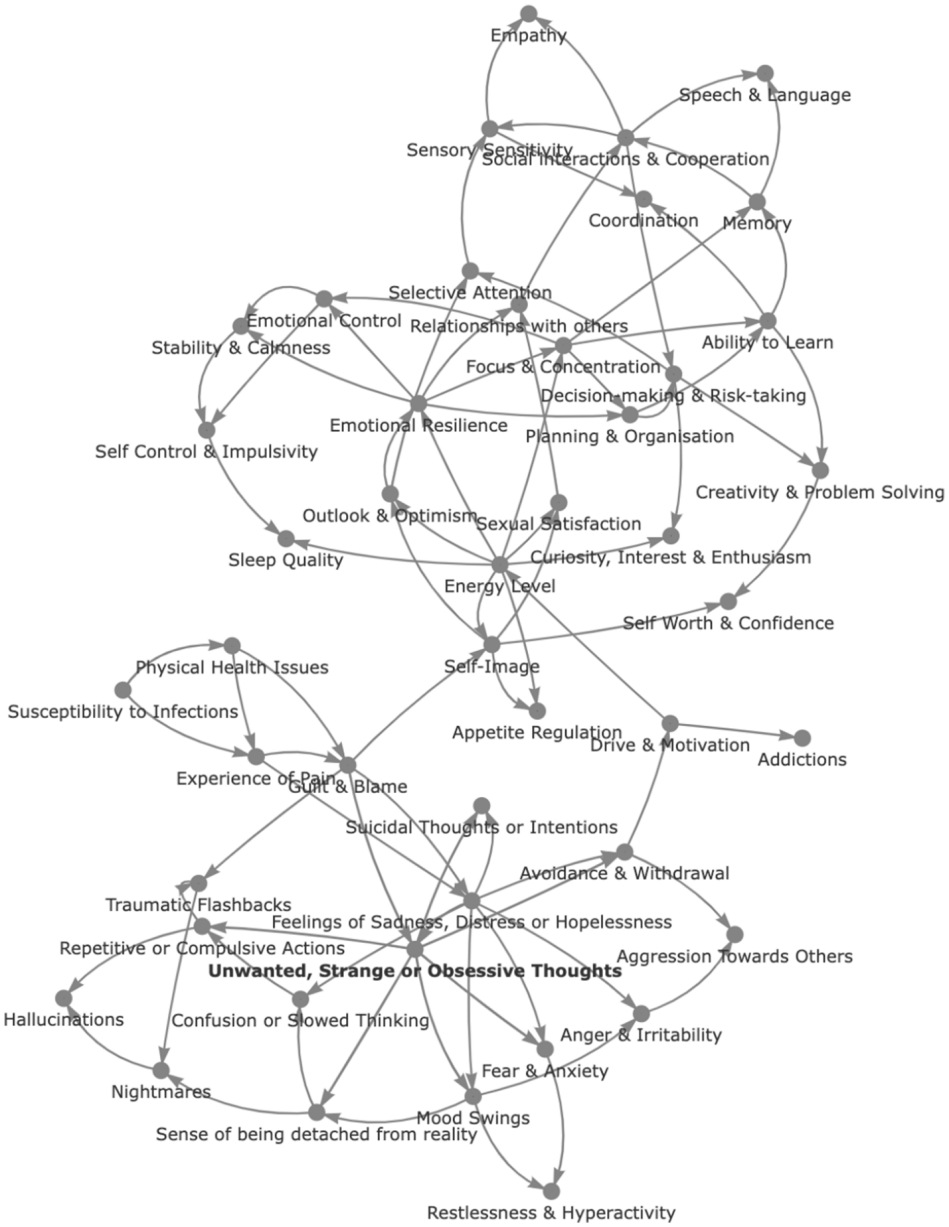
Causal inference graph of 47 symptoms across 270,000 records from the Global Mind data.

While such methods can uncover degree of importance, they do not provide insight into relative causality. Recent years have thus seen the emergence of causal graphical models (CGMs) or Bayesian networks as potent tools for modeling complex causal relationships (107) and unveiling the hierarchical structure of causal effects (108). These offer new possibilities for disentangling complex cause-and-effect relationships in observational data using directed acyclic graphs (DAGs) that represent causal relationships between variables, where nodes symbolize variables and edges indicate causal links. For illustrative purposes, we present in **Figure 6** a Bayesian network depicted as a causal inference graph, which prioritizes the hierarchical relationship among symptoms. This model applies Bayesian inference techniques to analyze 47 distinct symptoms across a dataset of 270,000 records from the Global Mind Project, spanning 70 countries, to elucidate relationships in the patterns of symptom manifestation. For example, in this graph, “Unwanted, strange, or obsessive thoughts” is a nodal symptom that appears to cause others such as “Fear and anxiety”, “Mood Swings”, “Sense of being detached from reality”, “Repetitive or compulsive actions” and “Avoidance and withdrawal”. So also “Focus & Concentration” has a nodal position with a causal path to “Ability to Learn”, “Memory”, “Planning and Organization” and “Emotional Control”. However, there are many nuances to this method and the strength of causality must be evaluated, which again can be done using various methodologies. While this example restricts analysis to the symptoms alone, application of causal inference to the Global Mind dataset with the inclusion of life context factors could similarly be used to uncover how substance use drives lifestyle behaviors and mental health outcomes, or how ultra-processed food consumption, life traumas and lifestyle factors interact to cause mental health symptoms.

While data from the ABCD Study has been available for several years already, we found the use of causal inference only in one paper, which looked at the relationship between prenatal cannabis exposure, sleep hours and internalizing problems (109). Thus, a host of possibilities remain unexplored. For example, comparing toxicity profiles obtained from biological samples with MRI metrics and behavioral measures such as UPPS-P for Children - Short Form [which assesses impulsivity (110);] and Prodromal Psychosis Scale [which assesses psychosis risk syndromes (111)], could provide a better understanding of the biological basis of certain behaviors. Within the ABCD data there is also the unique opportunity to look at causal trajectories over time. For example, when combining physiological measures and outcomes across subsequent years, how much do different life experiences at age 12 impact outcomes at age 17?

Like all data science applications, numerous other methods for identifying causal relationships are also available. Decision trees, when extended to causal relationships, estimate causal effects through Causal Tree-based Methods (112, 113), while Structural Causal Models (114) detail and simulate interventions based on explicit causal relationships and can predict the magnitude of changes in mental health outcomes due to various influences. Thus, multiple methods of causal exploration will have to be tried and differences in results debated. Finally, while such methods cannot definitively prove causality, they can provide a framework for identifying the most likely causal candidates that can then be tested in more rigorous studies.

### 4.3 Challenges and limitations

Big data approaches provide an important opportunity to explore a vast landscape of interacting factors to identify structures, patterns, and hierarchies. Furthermore, big data facilitates causal learning by exposing nuanced patterns not visible in small samples. Mining millions of records aids the discovery of subgroup-specific risks obscured by population averages. The demographic breadth of samples also enables the evaluation of assumptions and their robustness. For instance, testing regression consistency over various locations, periods, and subpopulations can identify relationships that are robust to culture. In addition, big data mitigates the risks of overfitting limited data.

However, there are also inherent limitations and cautions. These include data quality, completeness, structures, security and privacy and challenges of assumptions and interpretations.

*Data quality.* Causal discovery algorithms specifically model nonlinearity and stochasticity. In this context noisy inputs, therefore, propagate inaccuracies. Moreover, the assessment of symptoms is inherently subjective, contributing to the noise. Ensemble and aggregated modeling within the machine learning paradigm are designed to account for inherent variability by integrating multiple estimation approaches. In addition, debiasing training data and calculating missingness causally alleviates noise and incomplete information issues. However, there is no substitute for good quality data and rigorous evaluation and cleaning of data is essential. This can include utilization of internal checks and controls to determine if the same symptom queried in different ways has similar responses, if any values are out of plausible range, comparing outcomes across different sites or researchers involved in multi-site data acquisition to identify anomalies (as in the case of the ABCD Study) and comparing data across studies (e.g. Global Mind data to national surveys such as the American Community Survey or Household Pulse Survey) to check for consistency of overlapping variables.

*Completeness of the data*. The omission of important factors can provide an incomplete view of the data. Outcomes of clustering, predictive analysis and causal inference can all shift with the omission of a key nodal factor that is correlated to other factors considered. The opportunity of big data is thus enhanced by rapid exploration of new data points of relevance at large scale that allow iterative exploration. The Global Mind Project is specifically designed for such exploration by being an ongoing data acquisition program where the exploration of causal life factors can evolve with new hypotheses and ideas. In this paradigm, new questions can be swapped in and out and a hundred thousand records can be gathered in a few months.

A second aspect of data completeness is the sampling and whether it covers the breadth of populations that can enable a generalizable view. The Global Mind data for instance has wide global and demographic coverage where results can therefore be compared across language, country and ethnic groups. However, it is restricted to the internet-enabled audience and therefore not generalizable to the typically lower-income offline audience. In contrast, the ABCD Study is US specific and therefore does not allow cross-national comparisons. On the other hand, it recruits participants across a range of socio-economic groups and US geographies and enables a robust comparison within the country.

*Data Structures* A major challenge in working with big data is that of integrating across multiple data layers (e.g. symptom assessments and physiological metrics). Multiple data layers stored in different forms and associated with a single participant poses a considerable data management challenge and a barrier to effective use. Often integrating data layers can take more time than the analysis itself and researchers are loathe to spend their time on this exercise. Moreover, when the data is not properly annotated or documented it can lead to the introduction of considerable errors. Thus, a central goal of open data projects should be to develop data structures that are well annotated and easy to use whereby variables can be easily discovered and downloaded for analysis without substantial effort.

*Data security and privacy*. The power of large-scale data as we have described is best realized when it is open sourced for many researchers to use. However, aggregation and sharing of health-related information raises considerations relating to privacy, data-sharing and usage rights. Checks need to be in place to ensure that participants have appropriate rights over their personal data and that their data is only used in the manner outlined to the participant at time of consent. Attention should also be paid to data security to avoid breaches to participant privacy. In particular, identifiable information should be encrypted with encryption keys available only to a very small number of individuals who are bound by security protocols with periodic penetration testing conducted to identify and remedy any security weaknesses.

*Assumptions and interpretations of results.* While all machine learning algorithms are typically available as easy-to-use packages and code libraries, there are many nuances and choices that one must make in setting up the problem and processing the data. Thus, multiple perspectives on these problems are essential. This can pit methods and assumptions against one another to identify results that are robust to methods and data structures versus those that shift with methodology. Methods can then iteratively improve.

In summary, there are now both large datasets and powerful tools that can advance our understanding of multidimensional causal pathways with many-to-many interactions. Altogether this approach provides a framework for wide exploration and identification of the most likely causal candidates that can then be tested in more rigorous studies such as controlled, interventional studies. However, dataset characteristics and specific research objectives influence the choice of method which in turn may emphasize certain patterns and relationships while downplaying others. It is thus important to determine whether outcomes are consistent across methods as well as datasets, and reproducible by different research groups.

## 5 In conclusion

In conclusion, multidimensional data coupled with powerful analytical approaches have the potential to transform our definition and diagnosis of mental illness from symptom-based to one that is causal, enabling a revolution in psychiatric research and the prevention and treatment of mental health challenges.

However, the potential of these approaches depends crucially on the data. As in all large data exercises, wider breadth and larger scale of the data can contribute to deeper and more accurate insights by covering more relevant factors in the causal chain. Furthermore, the quality and accessibility of the data are paramount for research success. This includes easy understanding of open datasets and lower barriers to access and interpretation of the data through well-defined data dictionaries, database search tools and data structures that are easy to work with. In addition, framing of the questions and goals of any analysis effort, refining analytical methods, and translating findings into tangible improvements in mental healthcare are fundamental to the success of these efforts. Despite progress in computational psychiatry around precision approaches with a focus on tailored treatment regimens and efficacy prediction [e.g (30, 32)], there has been limited application of these approaches to multidimensional datasets such as the ABCD Study with a focus on prevention and causality. Establishing multi-disciplinary research teams with domain expertise spanning prevention psychiatry, sociology, computational science and data science would help to drive forward this research opportunity.

Altogether these large datasets and analytical toolkits now present the opportunity to untangle the many-to-many relationships between causal factors, physiology and symptoms and enable the development of strong hypotheses that can be tested in more controlled settings.

## Author contributions

JN: Conceptualization, Writing – original draft, Writing – review & editing. JB: Writing – original draft, Conceptualization, Writing – review & editing. JG: Conceptualization, Writing – original draft, Writing – review & editing. BM: Conceptualization, Writing – review & editing, Writing – original draft. TT: Conceptualization, Writing – original draft, Writing – review & editing.
